# (2*S*,4*S*)-2-[(*S*,*E*)-2-Bromo-1-nitro­methyl-3-phenyl­all­yl]-4-methyl­cyclo­hexa­none

**DOI:** 10.1107/S1600536812013098

**Published:** 2012-03-31

**Authors:** Long Zhao, Chao Wu, Wen-Zeng Weng, Chu-Xia Yan, Ai-Bao Xia

**Affiliations:** a State Key Laboratory Breeding Base of Green Chemistry-Synthesis Technology, Zhejiang University of Technology, Hangzhou 310014, People’s Republic of China; b Hangzhou Jiuyuan Gene Engineering Company Limited, Hangzhou 310014, People’s Republic of China

## Abstract

The crystal structure of the title compoud, C_17_H_20_BrNO_3_, contains three chiral centers, which all exhibit an *S* configuration. The C=C double bond has an *E* conformation. The cyclo­hexane ring is in a chair conformation. In the crystal, mol­ecules are linked by weak N—O⋯Br inter­actions [O⋯Br = 3.136 (4) Å].

## Related literature

For related compounds, see: Li *et al.* (2009 ▶); Wu *et al.* (2011 ▶). For the asymmetric Michael reaction, which allows for the formation of three contiguous asymmetric centers, see: Agarwal & Peddinti (2011 ▶); Lu *et al.* (2010 ▶); Luo *et al.* (2007 ▶).
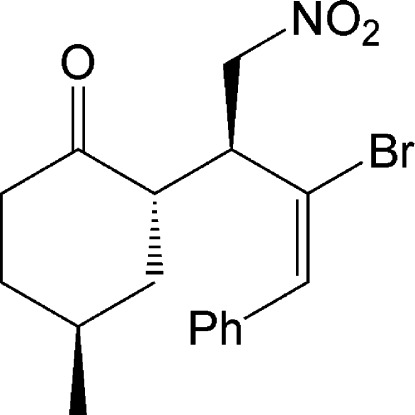


## Experimental

### 

#### Crystal data

C_17_H_20_BrNO_3_
*M* *_r_* = 366.25
Orthorhombic, 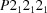

*a* = 7.0942 (5) Å
*b* = 13.7920 (11) Å
*c* = 17.3108 (13) Å
*V* = 1693.7 (2) Å^3^
*Z* = 4
Mo *K*α radiation
μ = 2.44 mm^−1^
*T* = 296 K
0.40 × 0.38 × 0.30 mm

#### Data collection

Rigaku R-AXIS RAPID/ZJUG diffractometer
Absorption correction: multi-scan (*ABSCOR*; Higashi, 1995 ▶) *T*_min_ = 0.377, *T*_max_ = 0.481
13310 measured reflections
3829 independent reflections
1967 reflections with *I* > 2σ(*I*)
*R*_int_ = 0.092

#### Refinement

*R*[*F* ^2^ > 2σ(*F* ^2^)] = 0.040
*wR*(*F* ^2^) = 0.106
*S* = 0.91
3829 reflections
200 parameters
H-atom parameters constrained
Δρ_max_ = 0.29 e Å^−3^
Δρ_min_ = −0.35 e Å^−3^
Absolute structure: Flack (1983 ▶), 1625 Friedel pairs
Flack parameter: −0.019 (14)

### 

Data collection: *PROCESS-AUTO* (Rigaku, 2006 ▶); cell refinement: *PROCESS-AUTO*; data reduction: *CrystalStructure* (Rigaku, 2007 ▶); program(s) used to solve structure: *SHELXS97* (Sheldrick, 2008 ▶); program(s) used to refine structure: *SHELXL97* (Sheldrick, 2008 ▶); molecular graphics: *ORTEP-3 for Windows* (Farrugia, 1997 ▶); software used to prepare material for publication: *WinGX* (Farrugia, 1999 ▶).

## Supplementary Material

Crystal structure: contains datablock(s) global, I. DOI: 10.1107/S1600536812013098/bh2424sup1.cif

Structure factors: contains datablock(s) I. DOI: 10.1107/S1600536812013098/bh2424Isup2.hkl

Supplementary material file. DOI: 10.1107/S1600536812013098/bh2424Isup3.cml

Additional supplementary materials:  crystallographic information; 3D view; checkCIF report

## Figures and Tables

**Fig. 1.**
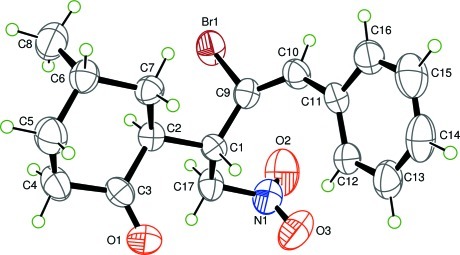
The asymmetric unit of the structure of the title compound, with displacement ellipsoids for non-H atoms at the 40% probability level.

**Fig. 2.**
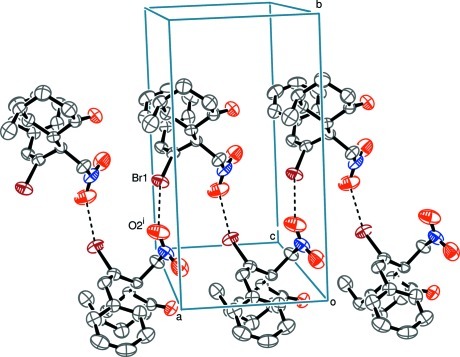
Unit cell packing of the title compound.

## supplementary crystallographic information

### Comment

Asymmetric Michael additions of aldehydes or ketones to nitroalkenes represent fundamental transformations which have wide applications in organic synthesis (Luo *et al.*, 2007; Lu *et al.*, 2010; Agarwal & Peddinti, 2011). On the other hand, alkenyl halides are present in a variety of natural products as well as in bioactive compounds. The title compound (Fig. 1) was obtained from the Michael addition of 4-methyl-cyclohexanone to (2-bromo-4-nitro-buta-1,3-dienyl)-benzene in our laboratory. The geometry compares well with other related structures (Li *et al.*, 2009; Wu *et al.*, 2011). In the title compound, the cyclohexyl ring adopts a chair conformation. The plane of the phenyl ring and the least-squares plane of the cyclohexyl moiety enclose an angle of 69.80 (3)°, while the plane through the nitro group and the adjacent C17 atom encloses an angle of 87.12 (3)° with the phenyl ring. The Br1—C9—C10—C11 torsion angle of 175.8 (4)° confirms the *E* configuration of the molecule with respect to the C9=C10 double bond. The molecules are linked by weak intermolecular N—O···Br interactions, the O···Br distance being 3.136 (4) Å.

### Experimental

A saturated brine (0.5 mL) solution of (2-bromo-4-nitrobuta-1,3-dienyl) benzene (1 mmol) and 4-methyl-cyclohexanone (1.2 mmol) was stirred with (*S*)-2-(pyrrolidin-2-ylmethylthio)pyridine (0.3 mmol) as catalyst and benzoic acid (0.3 mmol) as cocatalyst, at room temperature. After completion of the reaction, the mixture was extracted with ethyl acetate. Solvents were removed under vacuum and the residue was purified by column chromatography on silica gel (eluent: petroleum ether-ether). Suitable crystals were obtained by slow evaporation of an ethyl acetate solution.

### Refinement

H atoms were placed in calculated positions with C—H ranging from 0.93 to 0.98 Å and refined using riding model with *U*_iso_(H)=1.2*U*_eq_ or 1.5*U*_eq _of the carrier atoms.

### Crystal data

**Table:**

C_17_H_20_BrNO_3_	*F*(000) = 752
*M**_r_* = 366.25	*D*_x_ = 1.436 Mg m^−^^3^
Orthorhombic, *P*2_1_2_1_2_1_	Mo *K*α radiation, λ = 0.71073 Å
Hall symbol: P 2ac 2ab	Cell parameters from 8068 reflections
*a* = 7.0942 (5) Å	θ = 3.1–27.4°
*b* = 13.7920 (11) Å	µ = 2.44 mm^−^^1^
*c* = 17.3108 (13) Å	*T* = 296 K
*V* = 1693.7 (2) Å^3^	Chunk, colourless
*Z* = 4	0.40 × 0.38 × 0.30 mm

### Data collection

**Table:**

Rigaku R-AXIS RAPID/ZJUG diffractometer	3829 independent reflections
Radiation source: rotating anode	1967 reflections with *I* > 2σ(*I*)
Graphite monochromator	*R*_int_ = 0.092
Detector resolution: 10.00 pixels mm^-1^	θ_max_ = 27.4°, θ_min_ = 3.1°
ω scans	*h* = −9→7
Absorption correction: multi-scan (*ABSCOR*; Higashi, 1995)	*k* = −17→17
*T*_min_ = 0.377, *T*_max_ = 0.481	*l* = −22→21
13310 measured reflections	

### Refinement

**Table:**

Refinement on *F*^2^	Secondary atom site location: difference Fourier map
Least-squares matrix: full	Hydrogen site location: inferred from neighbouring sites
*R*[*F*^2^ > 2σ(*F*^2^)] = 0.040	H-atom parameters constrained
*wR*(*F*^2^) = 0.106	*w* = 1/[σ^2^(*F*_o_^2^) + (0.030*P*)^2^] where *P* = (*F*_o_^2^ + 2*F*_c_^2^)/3
*S* = 0.91	(Δ/σ)_max_ = 0.001
3829 reflections	Δρ_max_ = 0.29 e Å^−^^3^
200 parameters	Δρ_min_ = −0.35 e Å^−^^3^
0 restraints	Absolute structure: Flack (1983), 1625 Friedel pairs
0 constraints	Flack parameter: −0.019 (14)
Primary atom site location: structure-invariant direct methods	

### Fractional atomic coordinates and isotropic or equivalent isotropic displacement parameters (Å^2^)

**Table:**

	*x*	*y*	*z*	*U*_iso_*/*U*_eq_	
Br1	1.02011 (8)	0.36551 (3)	0.51179 (3)	0.0761 (2)	
O1	0.4791 (5)	0.6330 (2)	0.46539 (18)	0.0755 (8)	
O2	0.5698 (8)	0.3042 (3)	0.6070 (2)	0.1185 (17)	
O3	0.4316 (6)	0.4334 (4)	0.6423 (2)	0.1077 (15)	
N1	0.5198 (7)	0.3865 (3)	0.5965 (2)	0.0757 (12)	
C2	0.7862 (6)	0.5611 (3)	0.4626 (2)	0.0501 (11)	
H2	0.8173	0.5116	0.4240	0.060*	
C1	0.7353 (6)	0.5087 (3)	0.5385 (2)	0.0483 (11)	
H1	0.6865	0.5576	0.5744	0.058*	
C3	0.6255 (7)	0.6214 (3)	0.4309 (2)	0.0579 (12)	
C4	0.6690 (7)	0.6733 (4)	0.3565 (3)	0.0762 (15)	
H4A	0.5633	0.7141	0.3422	0.091*	
H4B	0.6880	0.6261	0.3156	0.091*	
C5	0.8469 (8)	0.7357 (4)	0.3653 (3)	0.0817 (17)	
H5A	0.8806	0.7624	0.3154	0.098*	
H5B	0.8197	0.7894	0.3997	0.098*	
C6	1.0146 (7)	0.6786 (3)	0.3972 (2)	0.0627 (11)	
H6	1.1128	0.7259	0.4101	0.075*	
C7	0.9604 (6)	0.6279 (3)	0.4720 (2)	0.0565 (10)	
H7A	0.9335	0.6764	0.5111	0.068*	
H7B	1.0663	0.5896	0.4899	0.068*	
C8	1.0995 (8)	0.6087 (4)	0.3388 (3)	0.0883 (18)	
H8A	1.2033	0.5748	0.3620	0.132*	
H8B	1.1435	0.6443	0.2947	0.132*	
H8C	1.0055	0.5629	0.3228	0.132*	
C9	0.9023 (7)	0.4616 (3)	0.5761 (2)	0.0563 (12)	
C10	0.9783 (7)	0.4757 (3)	0.6453 (2)	0.0599 (11)	
H10	1.0773	0.4343	0.6576	0.072*	
C11	0.9294 (7)	0.5474 (3)	0.7059 (2)	0.0537 (12)	
C12	0.7487 (7)	0.5782 (4)	0.7244 (2)	0.0666 (14)	
H12	0.6465	0.5530	0.6974	0.080*	
C13	0.7176 (8)	0.6451 (4)	0.7817 (3)	0.0798 (16)	
H13	0.5954	0.6654	0.7925	0.096*	
C14	0.8649 (10)	0.6819 (4)	0.8232 (3)	0.0842 (18)	
H14	0.8432	0.7277	0.8616	0.101*	
C15	1.0436 (9)	0.6515 (4)	0.8081 (3)	0.0895 (18)	
H15	1.1443	0.6762	0.8363	0.107*	
C16	1.0753 (7)	0.5830 (4)	0.7500 (2)	0.0720 (14)	
H16	1.1973	0.5611	0.7410	0.086*	
C17	0.5758 (6)	0.4353 (3)	0.5233 (2)	0.0625 (12)	
H17A	0.4679	0.4686	0.5014	0.075*	
H17B	0.6182	0.3871	0.4863	0.075*	

### Atomic displacement parameters (Å^2^)

**Table:**

	*U*^11^	*U*^22^	*U*^33^	*U*^12^	*U*^13^	*U*^23^
Br1	0.1030 (4)	0.0633 (3)	0.0619 (3)	0.0246 (3)	−0.0012 (3)	−0.0079 (2)
O1	0.067 (2)	0.076 (2)	0.083 (2)	0.016 (2)	0.0017 (18)	−0.0001 (17)
O2	0.175 (5)	0.079 (3)	0.101 (3)	−0.029 (3)	0.004 (3)	0.017 (2)
O3	0.105 (3)	0.132 (4)	0.086 (2)	−0.027 (3)	0.031 (2)	−0.021 (3)
N1	0.083 (3)	0.078 (3)	0.066 (2)	−0.029 (3)	0.000 (3)	−0.005 (2)
C2	0.060 (3)	0.041 (3)	0.049 (2)	−0.001 (2)	−0.001 (2)	−0.0039 (19)
C1	0.052 (3)	0.044 (3)	0.049 (2)	−0.002 (2)	−0.006 (2)	−0.0027 (19)
C3	0.057 (3)	0.058 (3)	0.059 (3)	−0.003 (3)	−0.014 (2)	−0.002 (2)
C4	0.084 (4)	0.078 (4)	0.067 (3)	0.004 (3)	−0.016 (3)	0.017 (3)
C5	0.089 (4)	0.077 (4)	0.079 (3)	−0.003 (3)	−0.001 (3)	0.022 (3)
C6	0.070 (3)	0.059 (3)	0.059 (2)	−0.010 (3)	0.002 (3)	0.003 (2)
C7	0.054 (3)	0.060 (3)	0.056 (2)	−0.004 (2)	0.002 (2)	−0.002 (2)
C8	0.092 (4)	0.105 (5)	0.068 (3)	−0.014 (3)	0.010 (3)	0.001 (3)
C9	0.066 (3)	0.052 (3)	0.051 (2)	−0.007 (2)	0.002 (2)	−0.001 (2)
C10	0.067 (3)	0.056 (3)	0.056 (2)	0.012 (3)	−0.005 (3)	0.0023 (19)
C11	0.065 (3)	0.054 (3)	0.042 (2)	0.001 (2)	−0.010 (2)	0.0020 (19)
C12	0.066 (4)	0.088 (4)	0.047 (3)	0.002 (3)	−0.003 (2)	−0.008 (2)
C13	0.103 (4)	0.084 (4)	0.052 (3)	0.017 (4)	−0.001 (3)	−0.011 (3)
C14	0.140 (6)	0.061 (4)	0.052 (3)	0.000 (4)	−0.001 (4)	−0.008 (3)
C15	0.116 (6)	0.089 (4)	0.064 (3)	−0.026 (4)	−0.016 (3)	−0.014 (3)
C16	0.073 (4)	0.082 (4)	0.061 (3)	−0.004 (3)	−0.005 (3)	0.003 (3)
C17	0.073 (3)	0.063 (3)	0.051 (2)	−0.020 (2)	−0.005 (2)	0.002 (2)

### Geometric parameters (Å, º)

**Table:**

Br1—C9	1.923 (4)	C7—H7A	0.9700
O1—C3	1.208 (5)	C7—H7B	0.9700
O2—N1	1.203 (5)	C8—H8A	0.9600
O3—N1	1.199 (5)	C8—H8B	0.9600
N1—C17	1.490 (5)	C8—H8C	0.9600
C2—C3	1.514 (6)	C9—C10	1.328 (5)
C2—C1	1.542 (5)	C10—C11	1.483 (6)
C2—C7	1.551 (5)	C10—H10	0.9300
C2—H2	0.9800	C11—C16	1.377 (6)
C1—C9	1.500 (6)	C11—C12	1.387 (6)
C1—C17	1.541 (6)	C12—C13	1.374 (7)
C1—H1	0.9800	C12—H12	0.9300
C3—C4	1.506 (6)	C13—C14	1.365 (8)
C4—C5	1.535 (7)	C13—H13	0.9300
C4—H4A	0.9700	C14—C15	1.361 (8)
C4—H4B	0.9700	C14—H14	0.9300
C5—C6	1.530 (6)	C15—C16	1.397 (7)
C5—H5A	0.9700	C15—H15	0.9300
C5—H5B	0.9700	C16—H16	0.9300
C6—C8	1.521 (6)	C17—H17A	0.9700
C6—C7	1.520 (5)	C17—H17B	0.9700
C6—H6	0.9800		
			
O3—N1—O2	124.3 (5)	C6—C7—H7B	109.1
O3—N1—C17	117.3 (5)	C2—C7—H7B	109.1
O2—N1—C17	118.4 (5)	H7A—C7—H7B	107.8
C3—C2—C1	112.9 (4)	C6—C8—H8A	109.5
C3—C2—C7	108.1 (3)	C6—C8—H8B	109.5
C1—C2—C7	112.0 (3)	H8A—C8—H8B	109.5
C3—C2—H2	107.8	C6—C8—H8C	109.5
C1—C2—H2	107.8	H8A—C8—H8C	109.5
C7—C2—H2	107.8	H8B—C8—H8C	109.5
C9—C1—C17	111.7 (4)	C10—C9—C1	130.5 (4)
C9—C1—C2	112.9 (3)	C10—C9—Br1	116.5 (4)
C17—C1—C2	109.5 (3)	C1—C9—Br1	113.0 (3)
C9—C1—H1	107.5	C9—C10—C11	129.8 (4)
C17—C1—H1	107.5	C9—C10—H10	115.1
C2—C1—H1	107.5	C11—C10—H10	115.1
O1—C3—C4	122.4 (4)	C16—C11—C12	117.2 (4)
O1—C3—C2	122.7 (4)	C16—C11—C10	117.0 (4)
C4—C3—C2	114.6 (4)	C12—C11—C10	125.7 (4)
C3—C4—C5	110.4 (4)	C13—C12—C11	121.3 (5)
C3—C4—H4A	109.6	C13—C12—H12	119.3
C5—C4—H4A	109.6	C11—C12—H12	119.3
C3—C4—H4B	109.6	C14—C13—C12	120.5 (5)
C5—C4—H4B	109.6	C14—C13—H13	119.8
H4A—C4—H4B	108.1	C12—C13—H13	119.8
C6—C5—C4	112.7 (4)	C15—C14—C13	119.8 (5)
C6—C5—H5A	109.0	C15—C14—H14	120.1
C4—C5—H5A	109.0	C13—C14—H14	120.1
C6—C5—H5B	109.0	C14—C15—C16	119.8 (5)
C4—C5—H5B	109.0	C14—C15—H15	120.1
H5A—C5—H5B	107.8	C16—C15—H15	120.1
C8—C6—C7	112.1 (4)	C11—C16—C15	121.3 (5)
C8—C6—C5	113.2 (4)	C11—C16—H16	119.4
C7—C6—C5	110.3 (4)	C15—C16—H16	119.4
C8—C6—H6	107.0	N1—C17—C1	110.3 (3)
C7—C6—H6	107.0	N1—C17—H17A	109.6
C5—C6—H6	107.0	C1—C17—H17A	109.6
C6—C7—C2	112.7 (3)	N1—C17—H17B	109.6
C6—C7—H7A	109.1	C1—C17—H17B	109.6
C2—C7—H7A	109.1	H17A—C17—H17B	108.1
			
C3—C2—C1—C9	−171.2 (3)	C17—C1—C9—Br1	63.8 (4)
C7—C2—C1—C9	−48.7 (5)	C2—C1—C9—Br1	−60.0 (4)
C3—C2—C1—C17	63.8 (5)	C1—C9—C10—C11	−4.4 (8)
C7—C2—C1—C17	−173.8 (3)	Br1—C9—C10—C11	175.8 (4)
C1—C2—C3—O1	5.7 (6)	C9—C10—C11—C16	−146.0 (5)
C7—C2—C3—O1	−118.9 (5)	C9—C10—C11—C12	37.3 (8)
C1—C2—C3—C4	−179.6 (4)	C16—C11—C12—C13	3.2 (7)
C7—C2—C3—C4	55.8 (5)	C10—C11—C12—C13	179.9 (5)
O1—C3—C4—C5	120.0 (5)	C11—C12—C13—C14	−1.1 (9)
C2—C3—C4—C5	−54.7 (6)	C12—C13—C14—C15	−0.7 (9)
C3—C4—C5—C6	52.3 (6)	C13—C14—C15—C16	0.3 (9)
C4—C5—C6—C8	73.0 (5)	C12—C11—C16—C15	−3.6 (7)
C4—C5—C6—C7	−53.5 (5)	C10—C11—C16—C15	179.4 (4)
C8—C6—C7—C2	−71.2 (5)	C14—C15—C16—C11	1.9 (8)
C5—C6—C7—C2	55.9 (5)	O3—N1—C17—C1	73.0 (5)
C3—C2—C7—C6	−56.0 (4)	O2—N1—C17—C1	−104.9 (5)
C1—C2—C7—C6	178.9 (4)	C9—C1—C17—N1	56.4 (5)
C17—C1—C9—C10	−115.9 (5)	C2—C1—C17—N1	−177.9 (4)
C2—C1—C9—C10	120.2 (5)		
